# Liver fibro-inflammation in psoriatic arthritis with MASLD improved by anti-IL-17 treatment: a clinical case

**DOI:** 10.1093/rheumatology/keaf177

**Published:** 2025-03-27

**Authors:** Lija James, Michele Pansini, Charlie Diamond, Hussein Al-Mossawi, Rajarshi Banerjee, Helena Thomaides-Brears, Laura C Coates

**Affiliations:** Nuffield Department of Orthopaedics, Rheumatology and Musculoskeletal Sciences, University of Oxford, Oxford, UK; Clinica Di Radiologia EOC, Istituto Di Imaging Della Svizzera Italiana, Ente Ospedaliero Cantonale, Lugano, Switzerland; Department of Radiology, John Radcliffe Hospital, Oxford University Hospitals NHS Foundation Trust, Oxford, UK; Perspectum Ltd, Oxford, UK; Nuffield Department of Orthopaedics, Rheumatology and Musculoskeletal Sciences, University of Oxford, Oxford, UK; Perspectum Ltd, Oxford, UK; Perspectum Ltd, Oxford, UK; Nuffield Department of Orthopaedics, Rheumatology and Musculoskeletal Sciences, University of Oxford, Oxford, UK

Rheumatology key message

- IL-17A inhibitor improved co-existing liver inflammation in a psoriatic arthritis patient with undiagnosed liver disease.

Dear Editor, We report a case demonstrating the reversal of liver fibro-inflammation through the use of a biologic DMARD (bDMARD) in a patient with psoriatic disease and undiagnosed liver disease. A 37-year-old woman with a 20-year history of psoriasis and 17 years of PsA was enrolled in the COLIPSO (Co-prevalence of liver disease in psoriatic disease) study, a long-term observational project investigating liver disease prevalence in psoriatic disease before and after treatment. The patient underwent quantitative multiparametric MRI (mpMRI) of the liver immediately before and after 6 months of treatment with secukinumab, an IL-17A inhibitor.

The patient had no previous history of fatty liver disease, diabetes, hypertension, or hyperlipidaemia. Her BMI was 32.7 kg/m^2^, and her alcohol intake was minimal (<5 units/week). Previous treatments for PsA included MTX (15 mg weekly), discontinued after 4 weeks in 2011 due to transaminitis, as well as LEF, adalimumab, and SSZ, all of which failed to control the disease.

At baseline, the patient’s DASs included 3 tender and swollen joints, CRP 3.6 mg/l and psoriasis skin body surface area of 3%. The Disease Activity in Psoriatic Arthritis (DAPSA) Score was 15.46, indicating moderate disease activity (a score within the range of 15 to 28). The 12-item Psoriatic Arthritis Impact of Disease (PsAID-12) is a patient-reported outcome measure that assesses the symptoms and impact of PsA, with a higher score indicating a poorer patient-reported status. The score obtained at baseline was 2.75 out of 10, suggesting moderate symptom/impact (a score within the range of >1.95 to ≤3.60). On clinical examination, there was no evidence of enthesitis, dactylitis, or axial SpA. The patient’s routine liver function tests were within normal limits [albumin 38 g/l (<50 g/l), bilirubin 10 µmol/l (<21 µmol/l), alanine transaminase 36 U/l (<40 U/l), and ALP 68 U/l (<130 U/l)]. An unexpectedly elevated Enhanced Liver Fibrosis (ELF) score of 8.4 indicated mild underlying early fibrosis. The ELF test is a non-invasive blood test used to assess the risk of advanced liver fibrosis in individuals with metabolic dysfunction-associated steatotic liver disease (MASLD). Furthermore, the initial mpMRI detected previously unidentified liver disease activity with significantly elevated inflammation levels. The iron-corrected T1 (cT1) score was measured at 901 ms (normal range <800 ms), and the liver fat (MRI-proton density fat fraction) was found to be 4.6%, close to the diagnostic threshold of ≥5% for MASLD (see Fig. 1).

**Figure 1.**
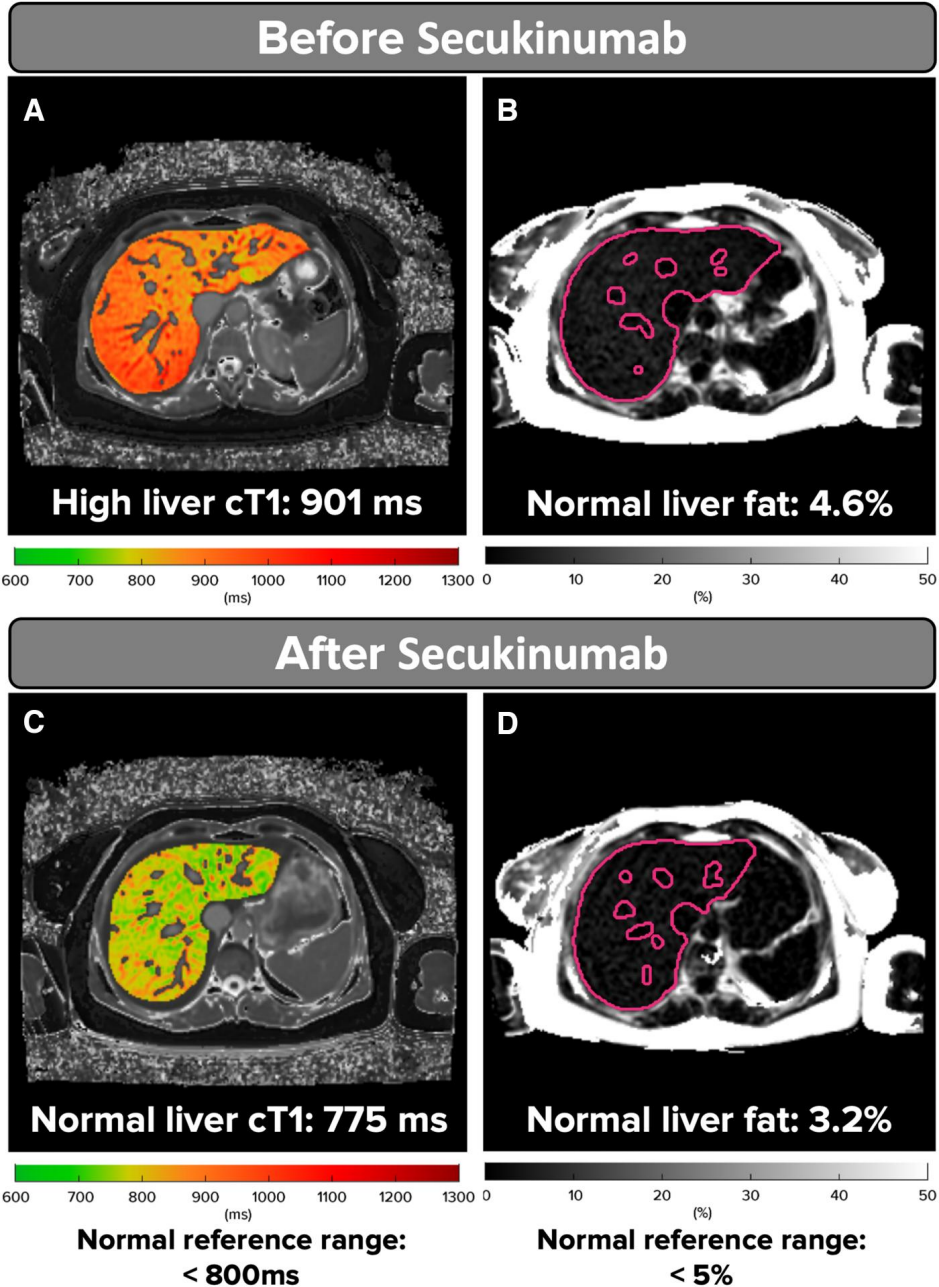
Case study of an individual with PsA demonstrating improvement in liver fibro-inflammation following secukinumab treatment. (A) Liver fibro-inflammation before treatment. (B) Liver fat content before treatment. (C) Liver fibro-inflammation after treatment. (D) Liver fat content after treatment. ^a^Iron-corrected T1 (cT1) measured in ms

After 6 months of secukinumab treatment (300 mg once monthly following the loading dose), liver metrics improved remarkably: cT1 decreased to 775 ms, MRI-PDFF (proton density fat fraction) to 3.2% (see Fig. 1), and the ELF score to 7.9. This reduction in cT1 is associated with a >2-point change in non-alcoholic fatty liver disease activity score, which is recognized in hepatology as a clinically meaningful improvement in liver health [1]. Clinical disease activity improved significantly, with zero tender and swollen joints, CRP of 1.3 mg/l, and psoriasis body surface area 0%. Additionally, the DAPSA score was 4.13 (a score of between 0 and 4 suggests PsA is in remission), and the PsAID-12 score was 0.75, a 2-point decrease for the PsAID-12 total score from baseline (a score of ≤1.15 denotes remission/no symptoms/no impact).

MASLD is estimated to be the leading cause of liver disease worldwide [2]. It is increasingly recognized as a common condition coexisting in patients with psoriatic disease, along with other comorbidities such as obesity and diabetes. Quantitative mpMRI, combining cT1 and MRI-PDFF, can rapidly and non-invasively quantify liver tissue characteristics, particularly inflammation and steatosis [3, 4].

In the context of psoriatic disease treatment, various biologic DMARDs target pro-inflammatory cytokines such as TNF alpha and ILs (IL-17 and IL-12/23). Nonetheless, there are currently no established guidelines for treating PsA with co-existing MASLD, and the precise impact of bDMARD medications on this condition is still unknown. Hepatic immune cell function undergoes restructuring in MASLD, which in turn plays a role in the development and progression of the disease [5]. Recent studies have highlighted the role of the type 3 cytokines, including IL-17 and IL-22, in modulating MASLD pathogenesis [5, 6]. Increased lipid uptake by liver cells and obesity drive higher IL-17 production, both systemically and within liver cells. IL-17 activates inflammation and biological processes, contributing to MASLD progression [7]. Gene expression profiling has further revealed a strong association between the IL-17 pathway and both psoriatic disease and MASLD [8]. These findings suggest a potential mechanistic link between the two conditions and highlight the possibility of targeting shared pathways for therapeutic benefit.

To our knowledge, this is the first reported case demonstrating the reversal of liver fibro-inflammation through the use of a biologic DMARD. This case suggests the potential of IL-17A inhibitors to improve co-existing liver disease in patients with PsA, underscoring the importance of considering liver health in psoriatic disease management. It suggests a possible dual benefit of certain biologic treatments, targeting shared immunological pathways between psoriatic disease and MASLD. Further research is warranted to explore the efficacy of biologic DMARDs in managing MASLD in patients with psoriatic disease, potentially opening new avenues for treatment strategies that address both conditions simultaneously.

Ethics committee approval was granted by Bromley REC for the COLIPSO study (Ref: 20/LO/0616). The patient in this case report has approved the publication and reviewed the report.

## Data Availability

The data underlying this article will be shared on reasonable request to the corresponding author.
